# Increases in statin eligibility to reduce cardiovascular risk according to the 2013 ACC/AHA cholesterol guidelines in the Africa Middle East region: a sub-analysis of the Africa Middle East Cardiovascular Epidemiological (ACE) study

**DOI:** 10.1186/s12872-019-1034-2

**Published:** 2019-03-15

**Authors:** Omar Hamoui, Mohamed I. Omar, Frederick J. Raal, Wafa Rashed, Abdoul Kane, Mohamed Alami, Paula Abreu, Walid Mashhoud, Alawi A. Alsheikh-Ali

**Affiliations:** 1 Cardiovascular Diseases, Clemenceau Medical Center, Beirut, Lebanon; 2 Medical Department, Pfizer Gulf FZ LLC, Dubai, United Arab Emirates; 3 Department of Medicine, Faculty of Health Sciences, University of the Witwatersrand, Johannesburg, South Africa; 4 Cardiology Division, Mubarak Al-kabeer Hospital, Jabriya, Kuwait; 5 Department of Cardiology, Dakar University, Hopital General de Grand yoff, Dakar, Senegal; 6 Private Practice, Casablanca, Morocco; 7 Pfizer Inc, New York, NY USA; 8 Pfizer Saudi Limited, Jeddah, KSA Saudi Arabia; 9 College of Medicine, Mohammed Bin Rashid University of Medicine and Health Sciences, Dubai, United Arab Emirates

**Keywords:** Africa Middle East region, Cholesterol guidelines, Lipid-lowering therapy, Income, The Africa and Middle East Cardiovascular Epidemiological (ACE) study, Statin therapy

## Abstract

**Background:**

With development of cholesterol management guidelines by the American College of Cardiology/American Heart Association (ACC/AHA), more individuals at risk of cardiovascular disease may be eligible for statin therapy. It is not known how this affects statin eligibility in the Africa and Middle East Region.

**Methods:**

Data were used from the Africa Middle East Cardiovascular Epidemiological (ACE) study. The percentage of subjects eligible for statins per the ACC/AHA 2013 cholesterol guidelines and the 2002 National Cholesterol Education Program-Adult Treatment Panel (NCEP-ATP III) recommendations were compared. Analyses were carried out according to age, gender, community (urban/rural), and country income categories based on World Bank definitions.

**Results:**

According to the ACC/AHA recommendations, 1695 out of 4378 subjects (39%; 95% confidence interval [CI], 37–40%) satisfied statin eligibility criteria vs. 1043/4378 (24%; 95% CI, 23–25%) per NCEP-ATP recommendations, representing a 63% increase in statin eligibility. Consistent increases in eligibility for statin therapy were seen according to the ACC/AHA vs. NCEP-ATP guidelines across sub-groups of age, gender, community, and country income. Notable increases for statin eligibility according to ACC/AHA vs. NCEP-ATP were seen, respectively, in subjects aged ≥65 years (86% vs. 39%), in males (46% vs. 25%), in low-income countries (28% vs. 14%), and rural communities (37% vs. 19%).

**Conclusion:**

An increase in statin eligibility was seen applying ACC/AHA cholesterol guidelines compared with previous NCEP-ATP recommendations in the Africa Middle East region. The economic consequences of these guideline recommendations will need further research.

**Trial registration:**

The ACE trial is registered under NCT01243138.

## Background

The use of lipid-lowering therapy, in particular statins, has been shown to reduce cardiovascular morbidity and mortality in numerous, multinational, randomized, double-blind clinical trials and meta-analyses [1–5]. Primary prevention trials in patients with cardiovascular risk factors have also demonstrated positive results with statin use, although with some variability in the magnitude of benefit [4, 6–8]. It is expected that increased use of lipid-lowering therapy in high-risk patients will result in wider benefits with short- and long-term use, leading to prevention of cardiovascular disease and the associated morbidity and mortality [9], sustaining a trend observed over the past few decades [10]. The socioeconomic implications of such trends are appealing, although this comes at a higher direct cost of prescription medications. Countries with adequate financial resources are better prepared to afford the short-term costs of prescription medications in order to recover the long-term benefits of reducing the morbidity and mortality associated with cardiovascular disease. Developing countries, on the other hand, with more limited financial resources, may be challenged to provide the medications and healthcare needed to meet recommendations for management of cardiovascular disease [11].

Developing countries share a large burden of the worldwide cardiovascular epidemic [12, 13]. Dyslipidemia constitutes a major risk factor for cardiovascular events in the Africa Middle East region [14–18]; for example, dyslipidemia is associated with the highest overall population-attributable risk among conventional risk factors for ischemic heart disease, according to the INTERHEART study, which included patients from Africa and the Middle East [12, 16]. Therefore, data on statin eligibility from developing countries are essential for adequate planning at the societal level.

Utilizing data from a study of cardiovascular risk factors across the Africa and Middle East region [15], the present study investigates the impact that 2013 ACC/AHA blood cholesterol guidelines [19] have on patient eligibility for statin use in this cohort of outpatients from the Africa Middle East region in comparison with the established National Cholesterol Education Program Adult Treatment Panel III (NCEP ATP III) blood cholesterol guidelines [20].

## Methods

Data collected for the Africa Middle East Cardiovascular Epidemiological (ACE) study [15] were analyzed for this sub-analysis. The ACE study evaluated cardiovascular risk factors of ambulatory patients attending general practice clinics in urban and rural areas of 14 countries in the Africa Middle East region. This multi-country, multicenter cross-sectional study was conducted between July 2011 and April 2012 [15]. Site selection was based on whether the centers had previous experience in clinical trials and whether ethical oversight and infrastructure were present for conducting clinical research [15].

Adult outpatients aged ≥18 years and who provided informed consent, were eligible for inclusion in the ACE study and for this sub-analysis. Every fifth outpatient attending the clinic for any reason was included in the study. Patients who were pregnant, lactating, or presented with a life-threatening illness were not eligible. The primary observation of the ACE study was percentage of outpatients with major cardiovascular risk factors, including dyslipidemia, hypertension, diabetes, obesity, and smoking [15]. Countries were selected based on the availability of epidemiologic data.

The site physician was responsible for the study evaluations, including history taking, physical examination, and laboratory tests. Laboratory evaluations were carried out during a single visit; a second visit was allowed only for non-fasting outpatients who returned to provide a fasting blood sample for the laboratory investigations. Ethics, institutional review board, and appropriate regulatory body approvals were obtained by all sites participating in the study. The primary results of the study have been previously reported [15]. The ACE study is registered on ClinicalTrials.gov identifier (NCT01243138).

The objective of the current sub-analysis was to estimate the percentage of outpatients in the ACE study population who would have been eligible for statin therapy according to the 2013 ACC/AHA guidelines [19] compared with the percentage who met eligibility criteria per the NCEP ATP III guidelines at enrollment [20]. The risk factors identified in the ACE study were used to ascertain the eligibility for statin therapy according to the above guidelines.

### Definitions of risk factors

Patients were identified as having dyslipidemia if they had at least one of: high total cholesterol, high low-density lipoprotein cholesterol (LDL-C), high triglycerides, low high-density lipoprotein cholesterol (HDL-C), or were on lipid-lowering/statin medications. High total cholesterol was defined as (≥240 mg/dL). Elevated LDL-C was defined as ≥100 mg/dL, ≥130 mg/dL, or ≥ 160 mg/dL in outpatients with high-, moderate-, or low cardiovascular risk, respectively, according to NCEP ATP III 2002 guidelines [20], which were contemporary and used in clinical practice at the time of the ACE study [15]. Low HDL-C level was defined as < 40 mg/dL in males and < 50 mg/dL in females. High triglyceride level was defined as ≥200 mg/dL. Additional risk factors assessed to determine cardiovascular risk status were: smoking; hypertension or on antihypertensive medication; low HDL-C (< 40 mg/dL); family history of premature coronary heart disease (CHD), defined as CHD in male first degree relative aged < 55 years, or CHD in female first degree relative aged < 65 years; and age (men ≥45 years; women ≥55 years). Diabetes was regarded as a CHD risk equivalent [20].

Hypertension was recorded in outpatients who were receiving anti-hypertensive medications or had a blood pressure (BP) measurement ≥140/90 mmHg, according to the European Society of Cardiology (ESC) guidelines (2007) [21], contemporary to the ACE study [15]. Diabetes mellitus was defined based on a fasting blood glucose measurement ≥126 mg/dL (7 mmol/L), per American Diabetes Association 2010 criteria [22] or the use of hypoglycemic agents. Former smokers were recorded as those who were not current smokers but had a history of smoking. The use of anti-hypertensive, lipid-lowering, and hypoglycemic therapy was also documented.

### Eligibility per ACC/AHA and NCEP ATP III

Patients were considered eligible for statin therapy per 2013 ACC/AHA guidelines if they had one of the following criteria: (1) clinical atherosclerotic cardiovascular disease (ASCVD); (2) primary elevations of LDL-C ≥ 190 mg/dL; (3) diabetes mellitus, with age 40–75 years, and with LDL-C 70–189 mg/dL in the absence of clinical ASCVD; or (4) LDL-C 70–189 mg/dL, with age 40–75 years, and with estimated 10-year ASCVD risk ≥7.5% in the absence of clinical ASCVD or diabetes mellitus. Outpatients aged 40–75 years, but without clinical ASCVD or diabetes, LDL-C 70–189 mg/dL, and with estimated 10-year ASCVD risk ≥5%, were also assessed. The 10-year risk for ASCVD was evaluated by using the Pooled Cohort Equations calculator [19].

Patients were considered eligible for statins per 2002 NCEP ATP III guidelines if they had one of the following: (A) 0–1 cardiovascular risk factors (per NCEP ATP III criteria, as outlined above) plus LDL-C ≥ 190 mg/dL; (B) 2 or more cardiovascular risk factors plus LDL-C ≥ 130 mg/dL; or (C) known CHD, transient ischemic attack, stroke, peripheral arterial disease, or diabetes plus LDL-C ≥ 100 mg/dL [20].

### Definitions of income status

The World Bank Atlas method was used to define the national income categories of the participating countries [23]. A country was classified as low income (LI) when the Gross National Income (GNI) per capita (US$) was $1005 or less in 2010. Countries with a GNI per capita of $1006 to $3975 were considered lower-middle income (LMI). Countries with a GNI per capita of $3976 to $12,275 were defined as upper-middle income (UMI), and countries with a GNI per capita of $12,276 or more were considered high income (HI). Income classification by country was as follows: *low income* – Kenya; *lower-middle income* – Cameroon, Ghana, Egypt, Nigeria, and Senegal; *upper-middle income* – Algeria, Jordan, Lebanon, Tunisia, and South Africa; *high income* – Kuwait, Saudi Arabia, and the United Arab Emirates.

### Definition of community

Rural areas were defined as areas greater than 50 km away from an urban area and without easy transportation [24].

### Statistical analysis

The number and percentage of outpatients for dichotomous variables, such as prevalence and number of outpatients who were eligible for lipid-lowering/statin therapy according to 2002 NCEP ATP III and 2013 ACC/AHA guidelines, are presented. Two-sided exact (Wilson Scores test) 95% confidence intervals (CIs) were used to calculate the percentage. Descriptive statistics, which included sample size, mean, standard deviation, median, minimum, and maximum, were used to summarize the continuous data (e.g., lipid parameters, BP parameters, and glucose). Outpatients were categorized by community (rural or urban), income level (LI, LMI, UMI, HI), gender and/or age range (young = aged 18–44 years, middle aged = 45–64 years, elderly = ≥65 years).

The full analysis set population served as the basis for all efficacy summaries and analyses, which were primarily descriptive in nature.

## Results

### Outpatient characteristics

The ACE study reported the prevalence of cardiovascular risk factors in 4378 outpatients [15]. The study population included 52% who were female and 31% from rural communities across the Africa Middle East region [15].

Of the total ACE study population, 197 (5%) outpatients had ASCVD, 136 (3%) had LDL-C ≥ 190 mg/dL; 510 (12%) had diabetes mellitus with no ASCVD and were aged 40–75 years with LDL-C 70–189 mg/dL; and 852 (20%) had an estimated 10-year ASCVD risk ≥7.5% per the Pooled Cohort Equation with no ASCVD or diabetes and aged 40–75 years with LDL-C 70–189 mg/dL (Table 1). Following the NCEP ATP III guidelines, 104 (2%) outpatients had 0–1 risk factor with LDL-C ≥ 190 mg/dL; 566 (13%) had 2 or more risk factors with LDL-C ≥ 130 mg/dL; and 373 (9%) had ASCVD or diabetes and LDL-C ≥ 100 mg/dL (Table 1). Overall, 1169 outpatients (27%) aged 40–75 years, but without diabetes or clinical ASCVD, had 10-year ASCVD risk ≥5% and LDL-C 70–189 mg/dL. Therefore, if a ≥ 5% ASCVD threshold was applied for statin eligibility in this population, rather than the ≥7.5% level, the total proportion of statin-eligible patients increases from 39% (1695/4378) to 46% (2012/4378).

**Table 1:** Statin-eligible groups by 2013 ACC/AHA and NCEP ATP III guidelines

Statin-eligible group	*n* (%)
per 2013 ACC/AHA guidelines	
Aged ≥21 y with ASCVD	197 (4.5)
Aged ≥21 y with untreated LDL-C ≥190 mg/dL	136 (3.1)
Aged 40–75 y with LDL-C 70–189 mg/dL and DM without ASCVD	510 (11.6)
Aged 40–75 y with LDL-C 70–189 mg/dL without ASCVD or DM and ASCVD risk ≥7.5%	852 (19.5)
Total eligible	1695 (39.0)
per 2002 NCEP ATP III guidelines	
0–1 risk factor and LDL-C ≥190 mg/dL	104 (2.4)
≥ 2 risk factors and LDL-C ≥130 mg/dL	566 (12.9)
CHD, TIA, stroke, PAD, DM, and LDL-C ≥100 mg/dL	373 (8.5)
Total eligible	1043 (24.0)

*ACC/AHA* American College of Cardiology/American Heart Association, *NCEP ATP III* National Cholesterol Education Program Adult Treatment Panel III, *ASCVD* atherosclerotic cardiovascular disease, *LDL-C* low-density lipoprotein cholesterol, *DM* diabetes mellitus, *CHD* coronary heart disease, *TIA* transient ischemic attack, *PAD* peripheral arterial disease

### Eligibility for statin therapy according to ACC/AHA or NCEP ATP III guidelines

**Fig. 1:**
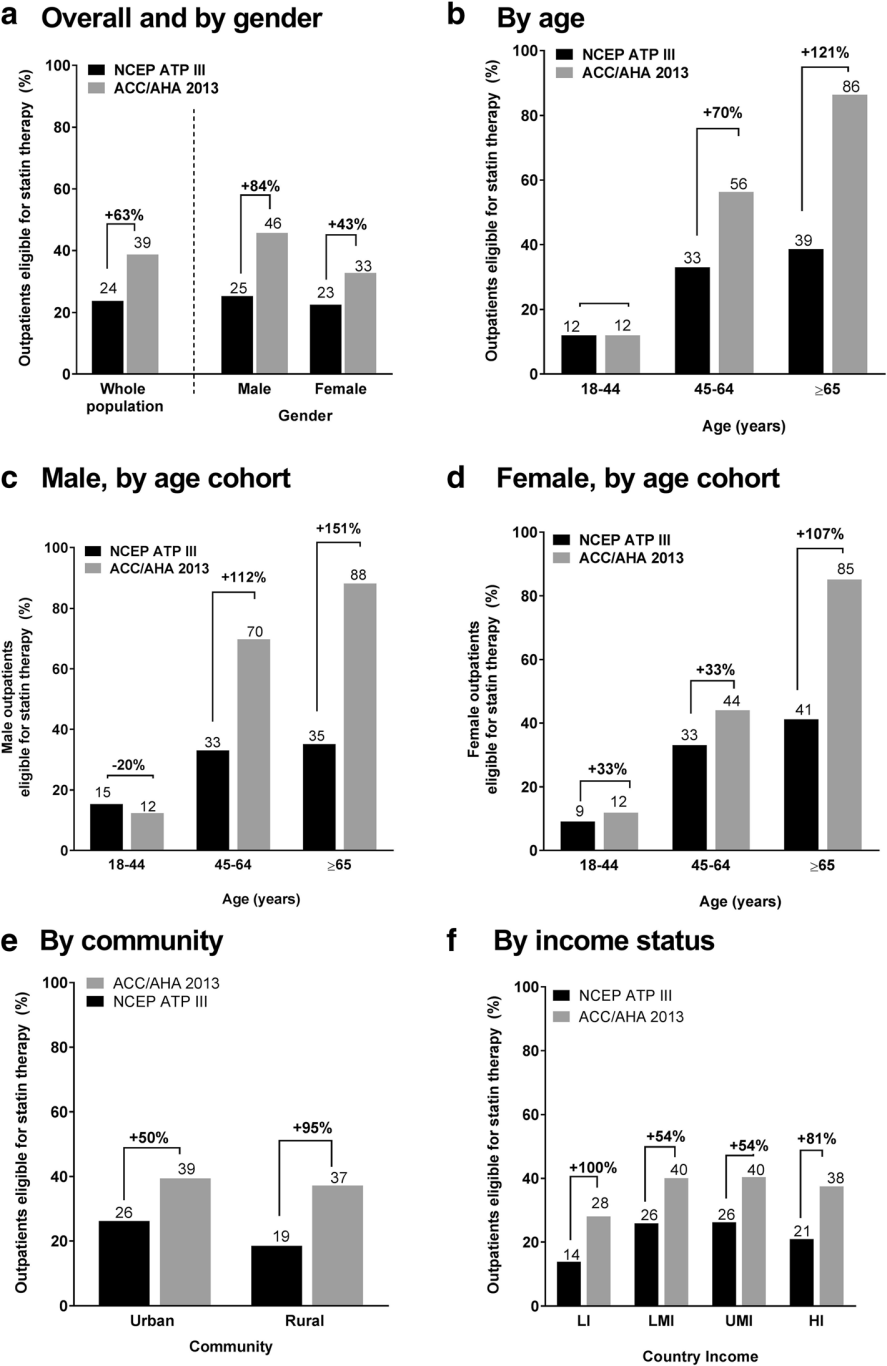
Statin eligibility by subgroups. *ACC/AHA* American College of Cardiology/American Heart Association, *NCEP ATP III* National Cholesterol Education Program Adult Treatment Panel III, *HI* high-income country, *LI* lower income country, *LMI* lower-middle income country, *UMI* upper-middle income country

A total of 1043 (24% [95% CI: 23, 25%]) outpatients were eligible for statin therapy per the NCEP ATP III guidelines and 1695 (39% [95% CI: 37, 40%]) were eligible according to 2013 ACC/AHA guidelines (Fig. 1). Most of the outpatients eligible for statin therapy per 2013 ACC/AHA guidelines qualified based on their calculated 10-year risk of ASCVD ≥7.5%, in the absence of clinical ASCVD or diabetes mellitus (20%); the remainder qualified based on having diabetes mellitus (12%), clinical ASCVD (5%), or primary elevations of LDL-C (3%) (Table 1).

Overall statin eligibility increased by 63% [(eligible per ACC/AHA) – (eligible per NCEP ATP III)/(eligible per NCEP ATP III)x100 = (1695–1043/1043) x100] according to the 2013 ACC/AHA guidelines compared with the earlier 2002 NCEP ATP III guidelines (Table 1; Fig. 1). This increase in eligibility for statin therapy was consistent across subgroups, namely male or female, urban or rural communities, age group (younger, middle-aged, older), and country income level (Fig. 1). When data were analyzed by age and gender, a similar pattern was seen across all age cohorts, with the exception of a slight reduction in young males (18–44 years) (Fig. 1c). Large increases in statin eligibility were seen for both elderly male and female outpatients, and in particular for male outpatients aged ≥65 years (+151% with NCEP vs. ACC/AHA) (Fig. 1c). Furthermore, according to the 2013 AHA/ACC guidelines, when ASCVD risk was set at ≥7.5%, the proportion of statin eligible patients showed a gender difference in the middle aged bracket (45–64 years), with a higher eligibility among men vs. women (70% [95% CI: 66.7, 72.7%) vs. 44% [95% CI: 40.9, 47.2%]) (Fig. 1c and d). According to the NCEP ATP III guidelines, this difference was not apparent, as the same proportion of male and female outpatients were eligible for statin therapy (33%) (Fig. 1c and d).

**Fig. 2:**
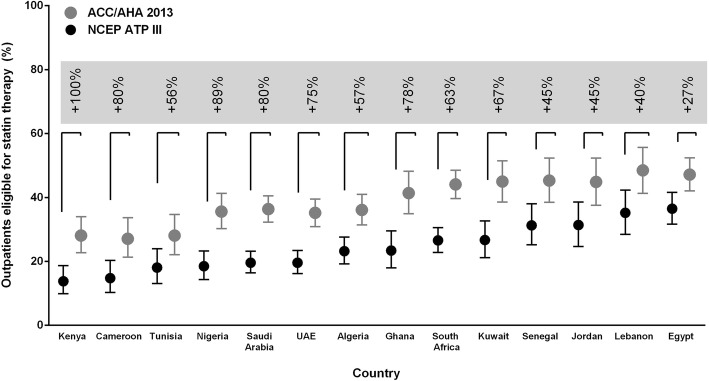
Statin eligibility by participating country: NCEP ATP III and the 2013 ACC/AHA criteria. The vertical lines represent 95% confidence intervals. The percentages at the top of the figure (gray boxes) represent the relative increase in the proportion of eligible patients per country. *ACC/AHA* American College of Cardiology/American Heart Association, *NCEP ATP III* National Cholesterol Education Program Adult Treatment Panel III, *UAE* United Arab Emirates

There were consistent increases in eligibility for statin therapy according to the 2013 ACC/AHA guidelines across the participating countries, ranging from a 27% increase in Egypt to 100% increase in eligibility in Kenya (Fig. 2). Significant increases in eligibility for statin therapy with 2013 ACC/AHA vs. NCEP ATP III guidelines, respectively, were also particularly noted among males (46% vs. 25%), in rural communities (37% vs. 19%), the elderly (aged ≥65 years) (86% vs. 39%), and low income countries (28% vs. 14%) (Fig. 1).

### Use of statin therapy according to NCEP ATP III guidelines

**Fig. 3:**
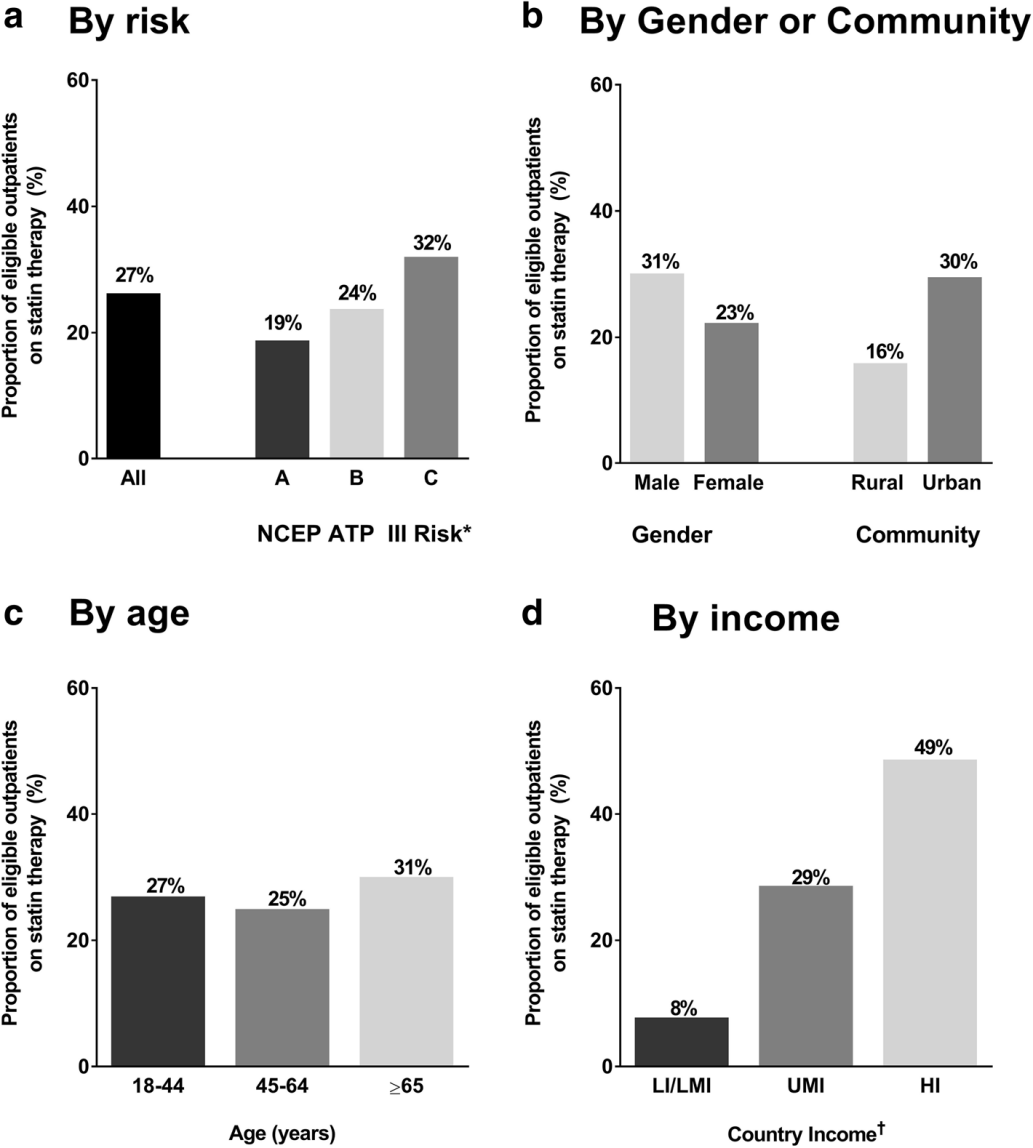
Proportion of eligible outpatients prescribed statins according to the NCEP ATP III guidelines. * NCEP ATP III indication: **a**, 0–1 risk factor plus LDL-C ≥ 190 mg/dL; **b**, ≥2 risk factors plus LDL-C ≥ 130 mg/dL; **c**, coronary artery disease, transient ischemic attack, stroke, peripheral arterial disease, diabetes mellitus, and LDL-C ≥ 100 mg/dL. Risk factors are listed in full in the Methods section. **d** † Country income levels: HI, high income; LI/LMI, lower/lower-middle income; UMI, upper-middle income. *NCEP ATP III* National Cholesterol Education Program Adult Treatment Panel III, *LDL-C* low-density lipoprotein cholesterol

Among outpatients eligible for statin therapy per NCEP ATP III guidelines [20], the use of these medications was low in the overall cohort (27%) (Fig. 3), including those with prior cardiovascular disease or risk equivalent (Group C, 32%; Fig. 3a). The lack of statin use in eligible outpatients was a consistent finding across gender, community (urban and rural), age groups, and by country income level. The proportion of statin-eligible outpatients prescribed medications was particularly low among women (23%), and those in rural communities (16%) or in lower-income (LI/LMI) countries (8%) (Fig. 3).

## Discussion

In a relatively young, ambulatory population attending general practice clinics in the Africa Middle East region, we observed a significant increase in eligibility for statin treatment when the 2013 cholesterol guidelines from ACC/AHA [19] were applied compared with the 2002 NCEP ATP III criteria [20]. Overall, there was a relative increase in statin eligibility of 63% (from 24 to 39%) in the overall study cohort, and an increase was observed in each of the 14 countries, ranging from a relative increase of 27% in Egypt to 100% in Kenya. This pattern was consistent across age groups, gender, community type, and national income levels. If the ≥5% ASCVD threshold was applied for statin eligibility, rather than the ≥7.5% level, the proportion of statin-eligible patients from the total study population increased even more, from 39 to 46%, with the highest proportion of patients (aged 40–75 years with LDL-C 70–189 mg/dL) being eligible based on predicted ASCVD risk ≥5%, but no diabetes or clinical ASCVD. This represents 7.2% of patients who had a predicted ASCVD ≥5 but <7.5%, who may be eligible for statin therapy, based on the presence of other cardiovascular risk factors contributing to the predicted risk, for example calcium scoring, ankle brachial index, or family history, etc. As noted above, more than one-third (39%) of the cohort had cardiovascular risk status making them eligible for statin therapy when the 2013 ACC/AHA statin therapy recommendations were applied. These individuals would be at high risk of developing future cardiovascular events, and would benefit significantly from intensive lifestyle management and statin therapy. This is supported by meta-analyses which have demonstrated that patients across different ethnicities, and at varying degrees of cardiovascular risk, benefit from statin therapy [3, 4, 25, 26], although with greater absolute benefits in patients at greater baseline risk [26].

The present findings are particularly relevant given the current burden and projected increase of cardiovascular disease morbidity and mortality in the developing world in general and within the Africa Middle East region in particular [27]. Developing countries are estimated to account for 85% of the global burden of cardiovascular disease [12, 13]. Conventional modifiable risk factors account for a large proportion of the cardiovascular disease burden in the Africa and the Middle East region [12, 16]. Notably, dyslipidemic factors are estimated to account for approximately 57 and 62% of the population attributable risk of myocardial infarction in the Middle East and Africa, respectively [12, 16]. In this context, assessing the proportion of adults eligible for statin therapy based on the cholesterol guidelines is relevant to healthcare providers and policy makers in their efforts to combat cardiovascular disease in these regions.

Increasing the number of outpatients eligible for statin therapy would have economic and system-wide implications. This is particularly true, as the most notable increase in statin eligibility in the present study was observed in lower-income countries with fewer resources, and in rural communities with potentially less-developed systems. Adherence to guidelines and the resulting increased eligibility for lipid-lowering therapy would require a significant increase in healthcare resources, as prescription drugs constitute a major element of healthcare costs of cardiovascular prevention. Judicious planning of healthcare resource utilization, improving access to care in rural communities, and coordinated efforts to lower prescription drug costs will be needed to meet the expected increase in eligible outpatients. The estimates provided by the present analysis should inform such efforts.

Approximately half of the outpatients eligible for statins per 2013 ACC/AHA guidelines qualified based on their calculated 10-year risk of ASCVD of ≥7.5% in the absence of clinical ASCVD or diabetes mellitus. These observations have important implications for the Africa Middle East region. The cost-effectiveness of statins in the primary prevention of cardiovascular disease is variable and is dependent on the estimated absolute risk of disease as well as the cost of prescription drugs. Both of these variables are not well-defined in the Africa Middle East region. Cost of drug data are not readily available and, given the variable cost structure and value of money, cost-effectiveness estimates from developed countries (United States or Europe) may not be directly applicable to the Africa Middle East region. Importantly, the risk model used to estimate risk of disease, upon which eligibility is reliant, has not been independently validated in cohorts from this region. There remains the potential for over-estimating disease risk with the ACC/AHA calculator when used for African or Middle Eastern patients, as has been observed in other cohorts [28, 29]. It is critical that such risk predictions, such as the ASCVD calculations, are validated in the Africa Middle East region if they are to be used for determining eligibility for life-long medications. Validation of such models is limited by the paucity of well-designed regional longitudinal studies with adjudicated cardiovascular outcomes. “Risk recalibration” for every country is necessary before using these scores. Developing countries can, as an alternative solution, use the World Health Organization (WHO) chart scores adapted for every country [30]. These models have also been validated.

An equally important observation of the current study is the under-prescribing of statins among patients who were eligible based on the NCEP ATP III criteria, which was the standard of care at the time of enrollment in the ACE study. Overall, three quarters of eligible patients were not on statin therapy, including two thirds of eligible high-risk patients with prior coronary artery disease, cerebrovascular disease, peripheral arterial disease or diabetes. This was most pronounced in lower/lower-middle income countries and rural communities where > 90 and > 80%, respectively, of eligible patients were not prescribed statins. Broadening the statin eligibility criteria with the newer guidelines together with low awareness and less education on the need for screening and treatment of dyslipidemia could further accentuate this pattern of under-prescribing among eligible outpatients in this population. Future studies are needed to identify barriers to adherence of guidelines in the Africa Middle East region, with a focus on understanding factors related to cost and access to healthcare.

The current analysis is strengthened by using a large dataset that includes outpatients from diverse countries and communities in Africa and the Middle East. Although we used standardized definitions of conventional risk factors, the study was limited by its cross-sectional design and reliance on single measurements. This may have affected the accuracy of our estimates, and all of our analyses should be considered descriptive in nature. Nonetheless, it is unlikely that these limitations would have differentially affected estimates from the criteria (NCEP ATP III or ACC/AHA 2013), and hence the observation of significant relative increases in eligible patients remains valid.

## Conclusions

Notwithstanding differences in local approved labels, our findings show that applying the 2013 ACC/AHA cholesterol guidelines significantly increased the number of statin-eligible adults attending outpatient clinics across the Africa Middle East region, particularly in the elderly men and women (aged ≥65 years), middle-aged men (aged 45–64 years), those from rural communities, and those from lower-income countries. Physicians should refer to individual product information for prescribing decisions. Further studies are needed to understand the feasibility and economic implications of these recommendations in the developing world.

## Abbreviations

ACC: American College of Cardiology
AHA: American Hypertension Society
CHD: coronary heart disease
CI: confidence interval
HDL-C: high-density lipoprotein cholesterol
LDL-C: low-density lipoprotein cholesterol
